# The Construction of a Prognostic Model Based on a Peptidyl Prolyl *Cis–Trans* Isomerase Gene Signature in Hepatocellular Carcinoma

**DOI:** 10.3389/fgene.2021.730141

**Published:** 2021-11-23

**Authors:** Huadi Shi, Fulan Zhong, Xiaoqiong Yi, Zhenyi Shi, Feiyan Ou, Yufang Zuo, Zumin Xu

**Affiliations:** Cancer Center, Affiliated Hospital of Guangdong Medical University, Zhanjiang, China

**Keywords:** peptidyl prolyl *cis*–*trans* isomerase, nomogram, hepatocellular carcinoma, TCGA, ICGC

## Abstract

**Objective:** The aim of the present study was to construct a prognostic model based on the peptidyl prolyl *cis*–*trans* isomerase gene signature and explore the prognostic value of this model in patients with hepatocellular carcinoma.

**Methods:** The transcriptome and clinical data of hepatocellular carcinoma patients were downloaded from The Cancer Genome Atlas and the International Cancer Genome Consortium database as the training set and validation set, respectively. Peptidyl prolyl *cis*–*trans* isomerase gene sets were obtained from the Molecular Signatures Database. The differential expression of peptidyl prolyl *cis*–*trans* isomerase genes was analyzed by R software. A prognostic model based on the peptidyl prolyl *cis*–*trans* isomerase signature was established by Cox, Lasso, and stepwise regression methods. Kaplan–Meier survival analysis was used to evaluate the prognostic value of the model and validate it with an independent external data. Finally, nomogram and calibration curves were developed in combination with clinical staging and risk score.

**Results:** Differential gene expression analysis of hepatocellular carcinoma and adjacent tissues showed that there were 16 upregulated genes. A prognostic model of hepatocellular carcinoma was constructed based on three gene signatures by Cox, Lasso, and stepwise regression analysis. The Kaplan–Meier curve showed that hepatocellular carcinoma patients in high-risk score group had a worse prognosis (*p* < 0.05). The receiver operating characteristic curve revealed that the area under curve values of predicting the survival rate at 1, 2, 3, 4, and 5 years were 0.725, 0.680, 0.644, 0.630, and 0.639, respectively. In addition, the evaluation results of the model by the validation set were basically consistent with those of the training set. A nomogram incorporating clinical stage and risk score was established, and the calibration curve matched well with the diagonal.

**Conclusion:** A prognostic model based on 3 peptidyl prolyl *cis*–*trans* isomerase gene signatures is expected to provide reference for prognostic risk stratification in patients with hepatocellular carcinoma.

## Introduction

The 2020 edition GLOBOCAN released by the World Health Organization shows that liver cancer ranks sixth in the number of new cases of malignant tumors worldwide and is the third leading cause of cancer death in the world (Sung et al., 2021). Hepatocellular carcinoma (HCC) is the most common pathological type of primary liver cancer, accounting for about 90% (Llovet et al., 2021). Current treatment options for HCC include radical hepatectomy, liver transplantation, arterial catheterization, radiotherapy, and chemotherapy. However, approximately 75% of patients are diagnosed with early disease after surgery relapse within 5 years. Moreover, surgical resection and liver transplantation are not appropriate for all HCC patients because most HCC patients are diagnosed as advanced or multifocal tumors, and the 5-year overall survival of HCC patients is less than 20% (Forner et al., 2018; Vibert et al., 2020; Yang and Heimbach, 2020). The TNM Classification of Malignant Tumors staging is one of the main reference indicators for prognosis assessment of HCC. However, TNM staging is insufficient in the assessment of prognosis due to the heterogeneity of tumors. The prognosis of HCC patients with the same TNM stage may vary, and even among HCC patients diagnosed with the same TNM stage and receiving similar clinical treatment, survival outcomes are various (Bruix et al., 2014; Dhir et al., 2016). Therefore, it is necessary to find more effective prognostic biomarkers in order to more accurately evaluate the prognosis and develop individualized treatment strategies.

Biological processes in the cell are extremely dynamic and complex events that are finely choreographed both spatially and temporally. The proper modulation of protein function is central to this orchestration. A number of regulatory mechanisms have been well-established, including post-translational chemical modifications of selected amino acid side chains, allosteric regulation, and regulated protein degradation (Lu et al., 2007). The peptidyl–prolyl *cis*–*trans* isomerases (PPIases) regulate the conversion between cis and trans conformations of proteins as a molecular timer and play an important regulatory role in the process of life activities (Lu et al., 2007). The PPIase superfamily comprises four structurally unrelated families: cyclophilins, FK506-binding proteins, parvulins, and the protein phosphatase 2A phosphatase activator. These proteins exhibit well-conserved CYP or FKBP domains. These four subfamilies of PPIases are not similar in their sequences and three-dimensional structures, but these proteins exhibit well-conserved CYP or FKBP domains and can all catalyze the *cis*–*trans* isomerism of the peptide–proline amide bond (Fischer et al., 1989; Fanghänel and Fischer, 2004; Jordens et al., 2006; Mueller and Bayer, 2008). Many members of the PPIases gene family have recently been found to be closely associated with cancer progression and prognosis (Hojo et al., 1999; Bao et al., 2004; Ni et al., 2010; Annett et al., 2020). However, the prognostic value of the PPIase gene signature in HCC remains unclear.

In this study, the transcriptome and clinical data of HCC patients were downloaded from TCGA and ICGC databases as training set and validation set, respectively. A prognostic model based on the PPIase gene signature was established by using Cox, Lasso, and stepwise regression methods. Kaplan–Meier survival analysis was used to evaluate the prognostic value of the model and validate it with an independent external data. In addition, nomogram and calibration curves were developed in combination with clinical staging and risk score.

## Methods

### Acquisition of Peptidyl Prolyl *Cis–Trans* Isomerase Gene Sets

We obtained 43 PPIase genes from the GO_PROTEIN_PEPTIDYL_PROLYL_ISOMERIZATION gene sets in the Molecular Signatures Database (MSigDB v7.2, http//: software.broadinstitute.org/gsea/msigdb) (Liberzon et al., 2011).

### Transcriptome and Clinical Data of Hepatocellular Carcinoma

Transcriptome and clinical data were downloaded from The Cancer Genome Atlas (TCGA, https://portal.gdc.cancer.gov/) (Blum et al., 2018). Information on the gene expression and comparing clinical data (377 cases; data format: BCR XML) were downloaded from the level 3 gene expression information (FPKM normalized) of the TCGA LIHC cohort. The data from TCGA were used as the training set, and the data from ICGC were used as the validation set. Another RNA-seq dataset of 240 primary HCC patients together with corresponding clinical information was accessed from the ICGC (https://dcc.icgc.org/, LIRI-JP) (Kennedy et al., 2012), which was used as a cohort for external validation of the signature. The clinicopathological data collected included sex, age, stage, grade, survival status, and survival duration in days. Our study was in accordance with the publication guidelines provided by TCGA.

### Identification of Differentially Expressed Genes

The differential expression of the PPIase gene in 370 HCC tissues and 50 para-cancerous tissues was analyzed by the “limma” package of R 3.6.1 software. The criteria for selection of differentially expressed genes were FDR <0.05, |log2FC|≥1, FDR: false discovery rate, and FC: fold change.

### Construction of the Prognostic Risk Score Model

The clinical data of HCC were merged with the expression data of PPIase genes. The “survival” package of R software was used to perform univariate Cox regression analysis. The hazard ratio (HR) and corresponding *p* value of each PPIase gene were obtained by univariate Cox regression analysis. When the *p* value was less than 0.05, the gene was selected for further analysis. In order to reduce the collinearity between genes and prevent the over-fitting of prognostic risk model variables, Lasso regression was used to further analyze the variables obtained from univariate Cox regression (Tibshirani, 1997). Subsequently, we performed further variable filtering through the “step” function, which was a stepwise regression analysis based on AIC information statistics. In addition, the coefficient of each PPIase gene was calculated by multivariate Cox regression analysis. Finally, the risk score equation was constructed as follows:

$$\mathrm{risk core=}\;\overset{n}{\underset{i=1}{\sum }}{\text{Coef}}_{\text{i}}\times X_{i},$$

where Coef is the coefficient, n is the number of genes, X is the expression value of the gene, and i refers to the serial number.

### Evaluation and Validation of the Prognostic Risk Score Model

The risk score of each HCC patient was calculated by the risk score equation. Patients were divided into low-risk and high-risk groups according to the median of risk score as the cutoff value. Kaplan–Meier survival analysis was performed using the “survival” package of R software. The “timeROC” package was used to draw the ROC curve of the model. The area under curve (AUC) was calculated to evaluate the sensitivity and specificity of the prognostic model. Principal component analysis was performed to explore the distribution pattern of high- and low-risk groups according to PPIase gene expression. In addition, we performed univariate and multivariate Cox regression analyses to investigate whether the risk score can be an independent predictor of overall survival in HCC patients. Covariates included age, stage, and grade. To verify the reliability of the model, we downloaded the LIRI-JP dataset from the ICGC database as the validation set. The risk score for each patient was calculated using the same formula as the training set.

### The Construction of Nomogram and Calibration Curves

In order to better evaluate the clinical significance of the model and facilitate clinical application, a nomogram integrating TNM staging and prognostic risk score was constructed. Clinicians can quantitatively assess the prognostic risk based on the score for each risk variable in the model. Finally, the calibration curve was drawn to evaluate the accuracy of the nomogram.

## Results

### Differential Expression of Peptidyl Prolyl *Cis–Trans* Isomerase Genes

**FIGURE 1:**
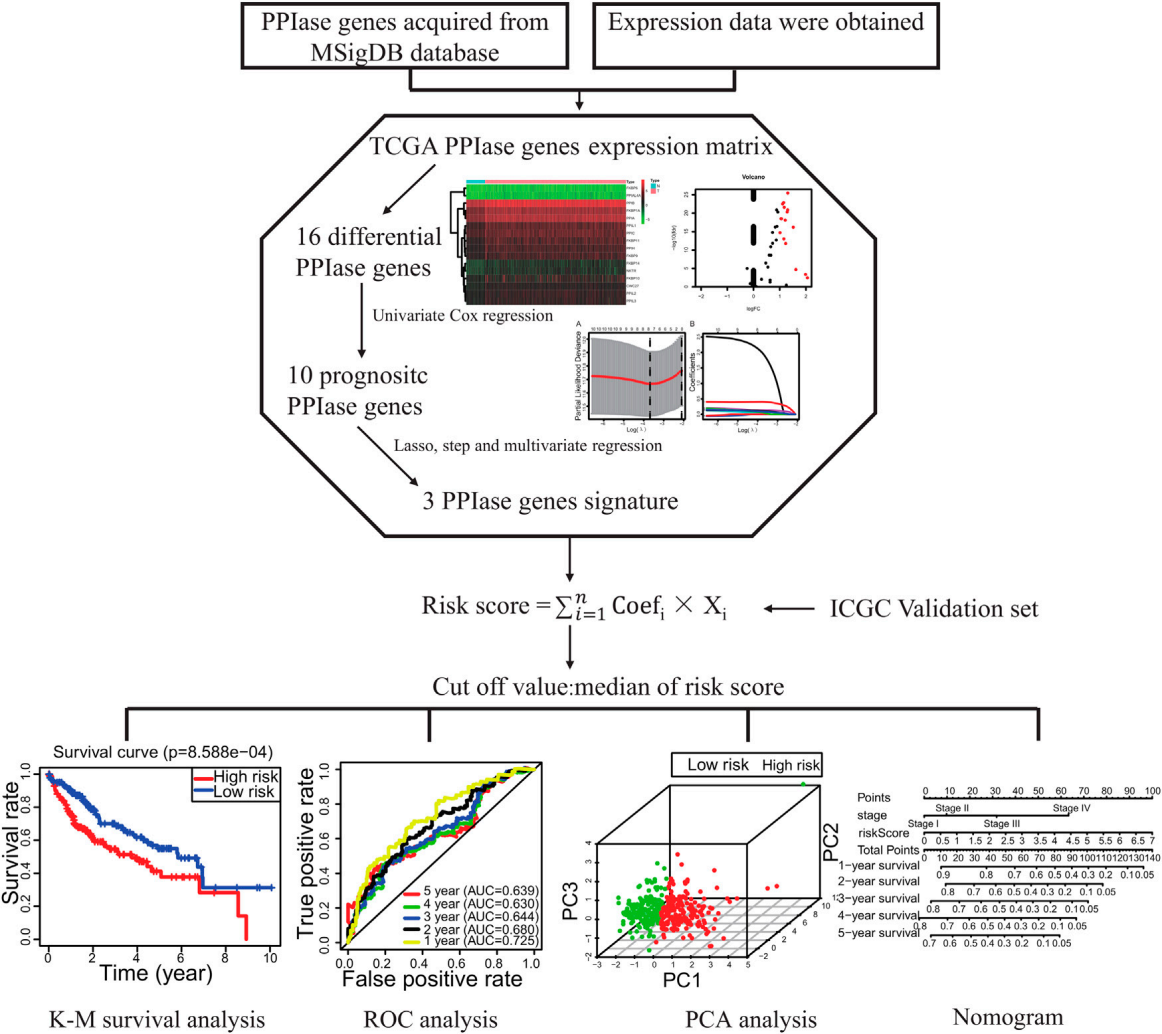
Flowchart for profiling the peptidyl prolyl *cis*–trans isomerase gene signatures.

**FIGURE 2:**
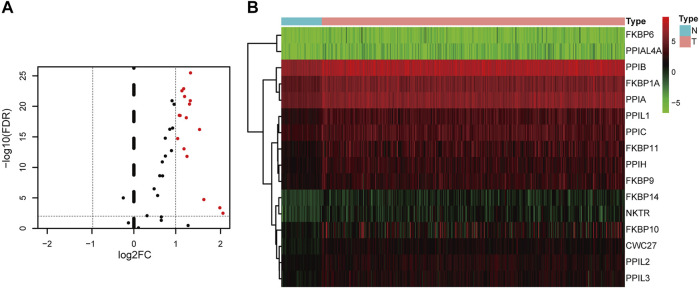
Differentially expressed PPIase genes. Volcano plot **(A)** and heat map **(B)** of differential expression in hepatocellular carcinoma and para-cancerous tissues.

Flowchart for profiling the PPIase genes of HCC (Figure 1). The TCGA–LIHC data were downloaded. There were 50 para-cancerous tissues and 370 HCC tissues which were included after data collection. The results showed that compared with the para-cancerous tissues, there were 16 upregulated PPIase genes in HCC tissues (Figures 2A,B).

### Construction of the Prognostic Risk Score Model Based on 3 Genes

**FIGURE 3:**
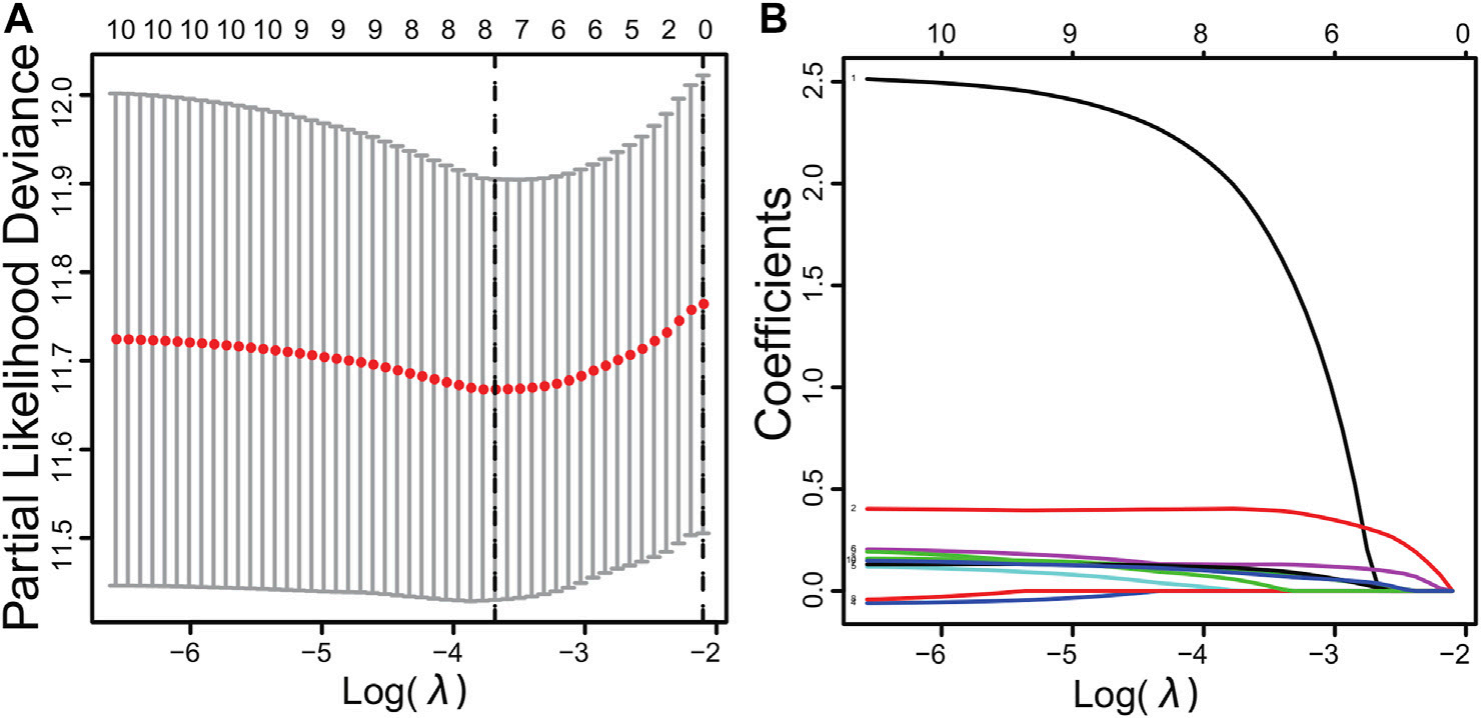
Cross-validation results **(A)** and dynamic process diagram of Lasso regression screening variables **(B)**.

Sixteen differentially expressed PPIase genes were included in univariate Cox regression analysis. There were 10 genes associated with survival in HCC patients, including FKBP6, CWC27, PPIH, FKBP10, PPIL2, FKBP1A, PPIL1, FKBP9, FKBP14, and PPIA (Table 1, *p* < 0.05). Lasso regression was applied to further screen the 10 prognostic PPIase genes, in order to reduce the influence of collinearity among genes and prevent over-fitting of risk model variables constructed later. The results of Lasso regression were included in the 7 PPIase genes: FKBP6, CWC27, PPIH, FKBP1A, PPIL1, FKBP14, and PPIA (Figure 3). Finally, a prognostic model based on the mRNA expression and coefficients of the 3 genes was finally obtained by multivariate Cox and stepwise regression analyses. The coefficients of each gene are listed in Table 2. The risk score was quantified by the following formula:

risk score=(2.68×FKBP6)+(0.67×CWC27)+(0.31×FKBP1A).

**TABLE 1:** Prognostic values of 16 PPIase genes.

Gene name	Regression coefficient	Hazard ratio (95%confidence interval)	*p*-value
FKBP6	3.227	25.2 (2.7–234.7)	**0.005**
CWC27	0.892	2.4 (1.6–3.6)	**0.000**
PPIB	0.010	1.0 (0.7–1.4)	0.950
FKBP11	0.152	1.2 (1.0–1.4)	0.118
PPIH	0.517	1.7 (1.3–2.2)	**0.000**
PPIAL4A	-0.446	0.6 (0.0–21)	0.802
FKBP10	0.125	1.1 (1.0–1.3)	**0.045**
PPIL2	0.372	1.5 (1.0–2.1)	**0.049**
FKBP1A	0.503	1.7 (1.3–2.1)	**0.000**
PPIL1	0.492	1.6 (1.2–2.1)	**0.000**
FKBP9	0.289	1.3 (1.0–1.7)	**0.022**
FKBP14	0.379	1.5 (1.0–2.1)	**0.031**
PPIA	0.582	1.8 (1.3–2.4)	**0.000**
PPIC	0.108	1.1 (0.9–1.4)	0.398
NKTR	0.094	1.1 (0.8–1.5)	0.539
PPIL3	0.002	1.0 (0.7–1.4)	0.993

Abbreviation: PPIase, peptidyl prolyl *cis*–trans isomerase. Bold values indicate *p* < 0.05.

**TABLE 2:** Most prognosis-related PPIase genes.

Gene name	Coefficient	Hazard ratio (95%confidence interval)	*p*-value
FKBP6	2.679,926	14.58 (1.36–156)	0.027
CWC27	0.669,348	1.953 (1.28–2.99)	0.002
FKBP1A	0.306,267	1.358 (1.01–1.83)	0.045

Abbreviation: PPIases, peptidyl prolyl *cis*–trans isomerases.

### Evaluation of the Peptidyl Prolyl *Cis–Trans* Isomerase Gene Signature Model

**FIGURE 4:**
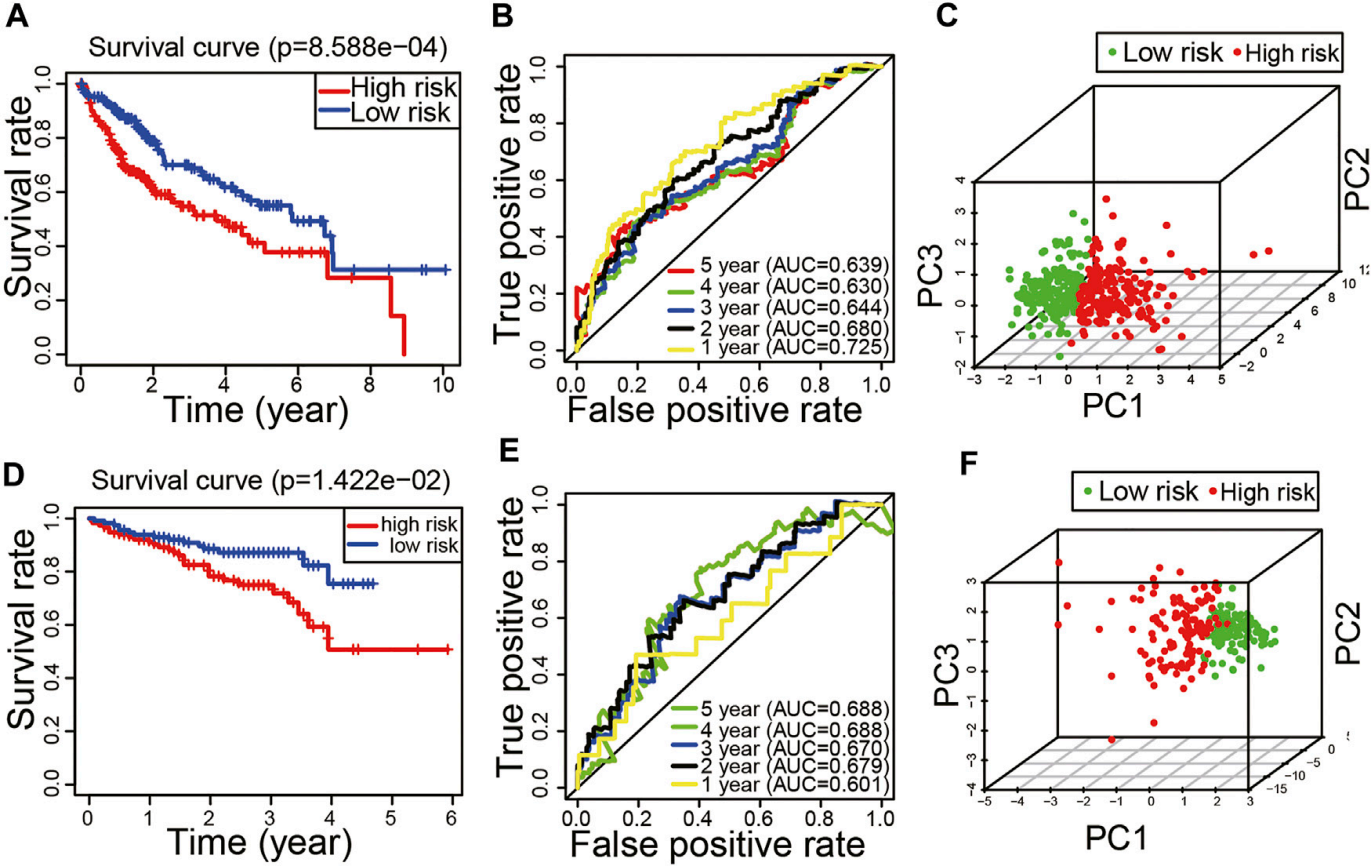
Evaluation and validation of the peptidyl prolyl cis–trans isomerase gene signature model. **(A)** Kaplan–Meier curve in the training set. **(B)** Time-dependent receiver operating characteristic curve in the training set. **(C)** Principal component analysis in the training set. **(D)** Kaplan–Meier curve in the validation set. **(E)** Time-dependent receiver operating characteristic curve in the validation set. **(F)** Principal component analysis in the validation set.

The Kaplan–Meier curve showed that HCC patients in high-risk score group had a worse prognosis (*p* < 0.05, Figure 4A). The ROC curve revealed that the AUC values of predicting survival rate at 1, 2, 3, 4, and 5 years were 0.725, 0.680, 0.644, 0.630, and 0.639, respectively (Figure 4B). The results of principal component analysis revealed that there were significant differences in the distribution patterns of HCC in the high-risk and low-risk groups (Figure 4C).

### Validation of the Peptidyl Prolyl *Cis-Trans* Isomerase Gene Signature Model

In order to verify the reliability of the model, we applied the external dataset from the ICGC database for validation. There were 230 HCC tissues which were included after data collation. The patients of the validation set were divided into high-risk (*n* = 115) and low-risk groups (*n* = 115) based on the median of risk score. Consistent with the results of the TCGA dataset, the Kaplan–Meier curve showed that HCC patients in high-risk score group had a worse prognosis (*p* < 0.05, Figure 4D). The ROC curve revealed that the AUC values of predicting survival rate at 1, 2, 3, 4, and 5 years were 0.601, 0.679, 0.67, 0.688, and 0.688, respectively (Figure 4E). The results of principal component analysis revealed that there were significant differences in the distribution patterns of HCC in the high-risk and low-risk groups (Figure 4F). It is suggested that the model has a good inclusiveness.

### Risk Score as an Independent Prognostic Factor

**FIGURE 5:**
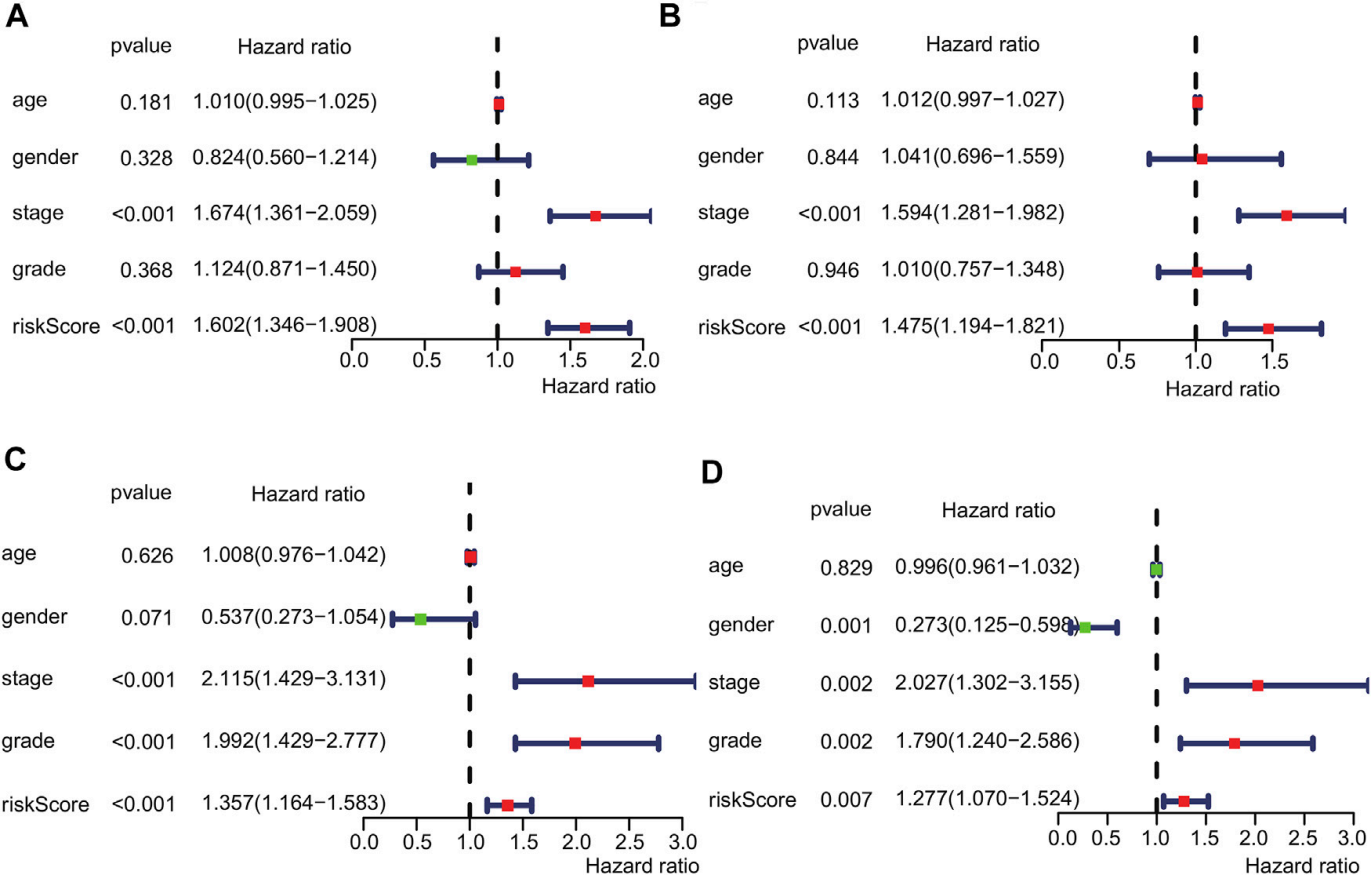
Risk score as an independent prognostic factor. Univariate **(A)** and multivariate **(B)** Cox regression analyses in the training set. Univariate **(C)** and multivariate **(D)** Cox regression analyses in the validation set.

Univariate and multivariate Cox regression analyses were performed to investigate whether the risk score could be an independent predictor of prognosis in patients with HCC. Univariate Cox regression analysis showed a significant correlation between the risk score and overall survival in the training set (HR = 1.602, 95% CI = 1.346–1.908, *p* < 0.001, Figure 5A). Multivariate Cox analysis suggested that the risk score was an independent prognostic predictor (HR = 1.475, 95% CI = 1.194–1.821, *p* < 0.001, Figure 5B). Similarly, univariate Cox regression analysis revealed that the risk score was related to overall survival in the validation set (HR = 1.375, 95% CI = 1.164–1.583, *p* < 0.001, Figure 5C). Multivariate Cox analysis suggested that the risk score was an independent prognostic predictor in the validation set (HR = 1.277, 95% CI = 1.070–1.524, *p* = 0.007, Figure 5D).

### The Construction of Nomogram and Calibration Curves

**FIGURE 6:**
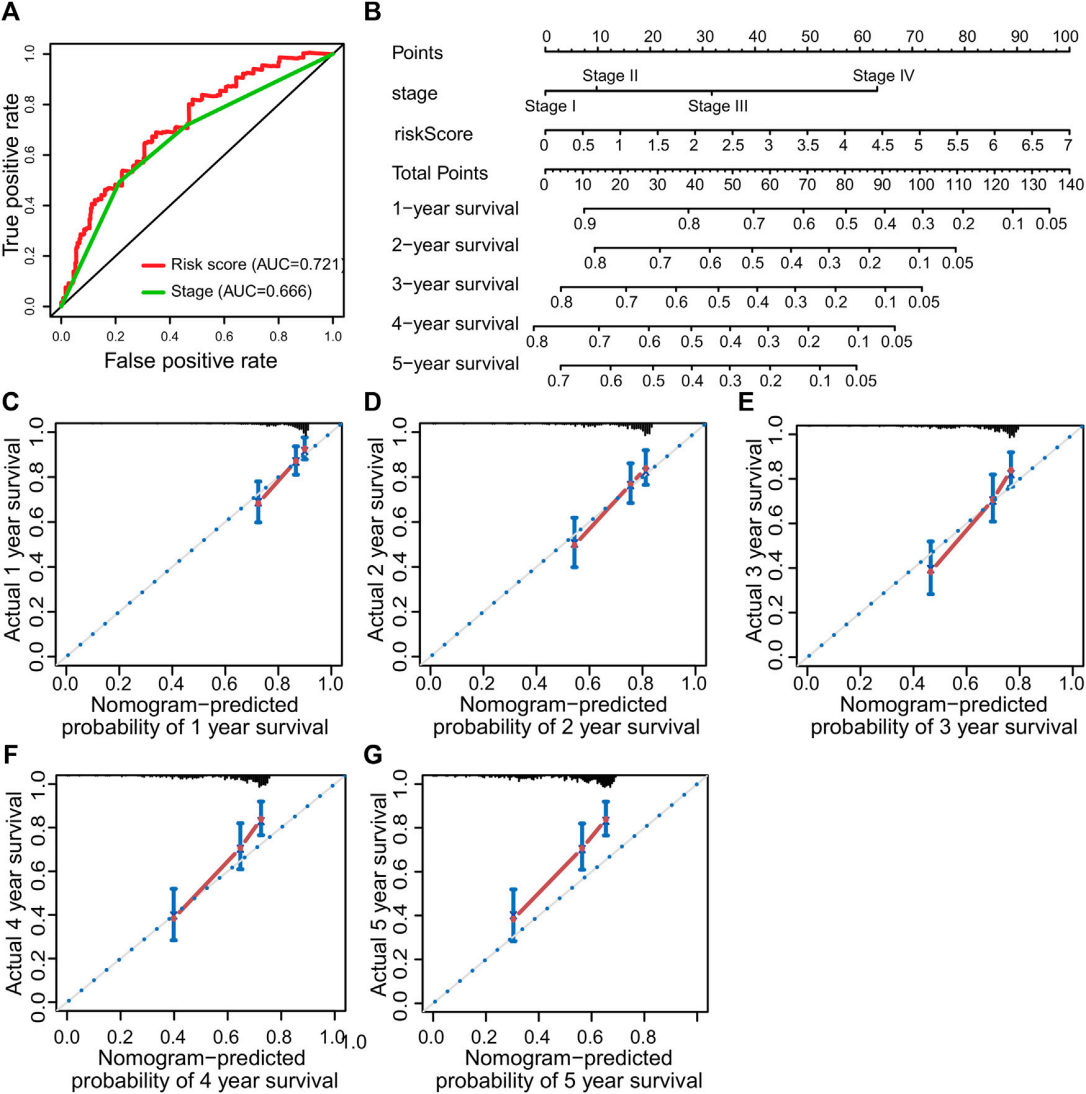
Prognostic nomogram was constructed to predict the overall survival probability based on the training set of patients with hepatocellular carcinoma. **(A)** ROC curve analysis. **(B)** The nomogram to predict 1-, 2-, 3-, 4- and 5-year OS overall survival in the train set. **(C–G)** The calibration plots for predicting patient 1-, 2-, 3-, 4- and 5-year OS, respectively.

The nomogram is a clinical tool that allows clinicians to determine the prognosis of patients by adding the score of each risk variable in the model to obtain the total score and the corresponding survival rate. Therefore, this study constructed a nomogram combining TNM staging and risk score. The ROC curve showed that the AUC value of risk score predicted 1-year survival was greater than stage (Figure 6A). The nomogram revealed that the risk score was the most important factor among the various clinical parameters (Figure 6B). Moreover, the 1-year, 2-year, 3-year, 4-year, and 5-year calibration curves have a high matching degree with the diagonal (Figures 6C–G).

## Discussion

In recent years, with the rapid development of next-generation sequencing technology and precision medicine, more and more evidence indicates that gene signatures of mRNA level have good potential in predicting the prognosis of many cancers, including HCC. For example, the application of bioinformatics methods to construct a prognostic model based on the gene signature of autophagy, M6A methylation, and immunity have been reported for a variety of cancers, which is even better than TNM staging to a certain extent (Brebi et al., 2014; Frost and Amos, 2017; Liu et al., 2021). However, most of the existing signatures have not been widely used in clinical practice of HCC because the reliability of models is affected by many factors such as over-fitting. In order to prevent over-fitting, some recent studies have adopted the regularization method, and the model has good reliability (Wang et al., 2020; Wang et al., 2021a; Li et al., 2021). Therefore, this study intends to use a combination of multiple regularization methods to construct an HCC prognosis model based on the PPIase gene set.

Many members of the PPIases gene family have recently been found to be closely associated with cancer progression and prognosis (Hojo et al., 1999; Bao et al., 2004; Ni et al., 2010; Annett et al., 2020). Therefore, we attempted to construct a prognostic model using the PPIases gene set. Surprisingly, we found that a model based on 3 PPIases gene signatures had good prognostic value. Multivariate Cox analysis suggested that the risk score was an independent prognostic predictor. The Kaplan–Meier curve showed that the prognosis of HCC patients in the high-risk group was worse. The AUC value of the ROC curve for predicting 1-year survival was greater than 0.7. A useful line nomogram was also successfully constructed.

In this study, PPIase gene differential expression was analyzed in HCC and adjacent tissues. Finally, 3 genes (FKBP6, CWC27, and FKBP1A) most related to prognosis were screened out by Cox and Lasso regression methods. It was reported that promoter methylation of FKBP6 can be used as a biomarker for the diagnosis of cervical cancer (Fischer et al., 1989). Another research showed that CWC27 can be used as a biomarker for the prognosis of bladder cancer (Wan et al., 2020). FKBP1A has also been reported to play a role in promoting tumor progression. Zhang et al. (2019) found that FKBP1A affected the proliferation and migration of prostate cancer cells (Lipunova et al., 2019). Romano et al. (2008) found that knockdown of FKBP1A can activate the TGF-β signaling pathway in chronic lymphocytic leukemia cells (Zhang et al., 2019). These studies suggest that FKBP1A may play a role in promoting cancer development in chronic lymphocytic leukemia and prostate cancer.

The nomogram is a clinical tool that allows clinicians to determine the prognosis of patients by adding the score of each risk variable in the model to obtain the total score and the corresponding survival rate (Romano et al., 2008). In recent years, the nomogram has been widely used as one of the practical tools in the assessment of cancer prognosis (Ohori Tatsuo Gondo And Riu Hamada et al., 2009; Zhou et al., 2021a; Wang et al., 2021b; Wu et al., 2021). Calibration curves are often used to evaluate the accuracy of a nomogram. The calibration curves of an ideal model just fall on the diagonal, and the more the calibration curves match the diagonal, the higher will be the prediction accuracy (Iasonos et al., 2008; Zhou et al., 2021b). As shown in Figures 5B,C,D, the calibration curve for predicting the survival rate at 1, 2, and 3 years has a good matching degree with the diagonal, suggesting a high accuracy of the model. Our model might provide a new reference for prognostic risk stratification assessment in HCC patients.

However, our model also has some limitations. First, further studies with additional external datasets are needed to confirm the prognostic value of the model. Second, the prognostic model is based on retrospective data, which is prone to selection bias, information bias, and other biases.

## Conclusion

A prognostic model based on 3 PPIase gene signatures is expected to provide reference for prognostic risk stratification in patients with HCC.

## Data Availability

The datasets presented in this study can be found in online repositories. The names of the repository/repositories and accession number(s) can be found in the article/Supplementary Material.
